# Does integration of HIV and sexual and reproductive health services improve technical efficiency in Kenya and Swaziland? An application of a two-stage semi parametric approach incorporating quality measures

**DOI:** 10.1016/j.socscimed.2016.01.013

**Published:** 2016-02

**Authors:** Carol Dayo Obure, Rowena Jacobs, Lorna Guinness, Susannah Mayhew, Integra Initiative, Anna Vassall

**Affiliations:** a Department of Global Health and Development, Faculty of Public Health and Policy, London School of Hygiene and Tropical Medicine, 15-17 Tavistock Place, London, WC1H 9SH, UK; b Centre for Health Economics, The University of York, Heslington, York, YO10 5DD, UK

**Keywords:** Kenya, Swaziland, Technical efficiency, HIV, Sexual and reproductive health, Data envelopment analysis, Semi-parametric, Quality of care

## Abstract

Theoretically, integration of vertically organized services is seen as an important approach to improving the efficiency of health service delivery. However, there is a dearth of evidence on the effect of integration on the technical efficiency of health service delivery. Furthermore, where technical efficiency has been assessed, there have been few attempts to incorporate quality measures within efficiency measurement models particularly in sub-Saharan African settings.

This paper investigates the technical efficiency and the determinants of technical efficiency of integrated HIV and sexual and reproductive health (SRH) services using data collected from 40 health facilities in Kenya and Swaziland for 2008/2009 and 2010/2011. Incorporating a measure of quality, we estimate the technical efficiency of health facilities and explore the effect of integration and other environmental factors on technical efficiency using a two-stage semi-parametric double bootstrap approach.

The empirical results reveal a high degree of inefficiency in the health facilities studied. The mean bias corrected technical efficiency scores taking quality into consideration varied between 22% and 65% depending on the data envelopment analysis (DEA) model specification. The number of additional HIV services in the maternal and child health unit, public ownership and facility type, have a positive and significant effect on technical efficiency. However, number of additional HIV and STI services provided in the same clinical room, proportion of clinical staff to overall staff, proportion of HIV services provided, and rural location had a negative and significant effect on technical efficiency.

The low estimates of technical efficiency and mixed effects of the measures of integration on efficiency challenge the notion that integration of HIV and SRH services may substantially improve the technical efficiency of health facilities. The analysis of quality and efficiency as separate dimensions of performance suggest that efficiency may be achieved without sacrificing quality.

## Highlights

• Substantial technical inefficiency found across facilities studied.
• Additional HIV/STI services in the MCH unit may improve technical efficiency.
• Functional integration has no significant effect on technical efficiency.
• Majority of the facilities exhibit low technical efficiency and low quality of care.
• Technical efficiency may be achieved without sacrificing quality.

## 1 Introduction

Integration of HIV and sexual and reproductive health services (SRH) has been widely adopted in resource constrained high HIV prevalence settings. In addition to improving health and social outcomes, integration of HIV and SRH services has been argued to hold the promise of increasing both cost and technical efficiency of service delivery and thereby maximizing the use of scarce health care resources (Dudley and Garner, 2011). Indeed, economic theory suggests several potential efficiency advantages at both the programmatic and service delivery levels from the integration of HIV and SRH services (Ickovics, 2008). However, despite the well-articulated economic rationale for integrating these services, evidence on the efficiency of integrated HIV and SRH services remains scarce.

In this context, integration is defined as the provision of two or more services at the same facility, with the service provider actively encouraging clients to use the other services in the same facility during the same visit. Within the literature, integration has been typically defined as either structural or functional integration (Sobczak, 2002). Structural integration is characterized by the provision of comprehensive services under one roof or within the same facility. Functional integration also entails coordination of health care activities and functions within a single complex organization. However, unlike structural integration, functional integration examines the extent to which the client is able to receive multiple services from the same provider (Kisubi et al., 1997).

As health care costs continue to rise and human resource constraints intensify, increasing the technical efficiency of healthcare delivery continues to be a subject of debate in high HIV prevalence settings. Moreover, the current uncertainty about the capability of developed countries to meet their international commitments to fund health programs in developing countries (Murray et al., 2011), has further intensified interest in integration as a means to improve the efficiency of HIV programs and thus reduce the resource needs of the HIV response. Many countries in sub- Saharan Africa (SSA) have already integrated HIV with SRH services and the most recent UNAIDS report (UNAIDS, 2013) notes a clear trend towards integration of HIV services with a number of countries addressing integration in their national health strategic plans (UNAIDS, 2013).

While improving efficiency is a valid public health aim, ensuring quality of care is also critical; and there is concern that when resources are constrained, the quality of care may be compromised. However, despite the importance of quality as a key element of performance in the health sector, there have been few attempts to date to incorporate quality measures within efficiency measurement models in low and middle-income (LMIC) contexts (Au et al., 2014). This is in part due to significant methodological challenges, not only related to the measurement of quality indicators, but also how to incorporate aspects of quality of care into the efficiency measurement framework (Hollingsworth, 2008).

Broadly, three main approaches have been used within the literature to incorporate service quality into efficiency evaluation using Data Envelopment Analysis (DEA) techniques. First, assuming that quality affects the efficiency of service delivery, some studies have included quality measures as additional exogenous variables in a second stage analysis (Nyman et al., 1990). Second, an alternative approach within the efficiency measurement literature has been to incorporate quality measures directly into the standard efficiency measurement model as an additional output (Cordero Ferrera et al., 2013, García et al., 1999, Nedelea and Fannin, 2013, Rosenman and Friesner, 2004, Salinas-Jiménez and Smith, 1996, Wagner et al., 2003). Third, quality and efficiency have been considered as separate dimensions and decision making units mapped in terms of their efficiency and quality scores (Sherman and Zhu, 2006).

In light of these considerations, the objectives of this paper are twofold. First, it aims to use recent methodological advancements in healthcare efficiency measurement to estimate technical efficiency of integrated HIV and SRH services and determine the effect of integration on technical efficiency of health facilities using data from Kenya and Swaziland. Specifically, a two stage semi-parametric approach with a double bootstrap procedure is implemented. In the first stage, DEA - a non-parametric technique employed widely in efficiency measurement – is used to estimate relative technical efficiency of health facilities while controlling for quality of the health services. Quality of the health services is measured using structural, technical and interpersonal aspects of quality of care. These quality dimensions ascertain the functional ability of facilities to provide services of acceptable standards, how well knowledge is applied to the diagnosis and treatment of medical problems, and the interaction between the provider and the client.

In the second stage, the efficiency scores are regressed against a set of explanatory variables expected to influence technical efficiency of health facilities. This formulation assumes that the explanatory variables influence the efficiency with which inputs generate outputs but do not influence the production process itself (Arocena and García-Prado, 2007).

Second, we explore alternative treatments of the quality measures as input or output variables incorporated into the standard efficiency measurement model. The results of the different DEA model specifications are assessed to determine how model specification affects the estimated technical efficiency of health facilities. In addition, we also consider quality and efficiency as two separate performance dimensions to examine the existence of a potential trade-off between quality and efficiency.

The paper is organized as follows. Section 2 outlines the methodological approach employed to analyze technical efficiency and incorporate quality and explanatory variables into the efficiency measurement framework. Section 3 describes the data available and the variables used in the study. Section 4 presents the results. Section 5 presents a discussion, some policy and practice implications and concludes.

## 2 Methods

Technical efficiency is defined as the ability of a production unit (referred to as a decision making unit (DMU)) to produce maximum output from a given level of inputs. DEA, a linear programming methodology introduced by Charnes, Cooper and Rhodes (Charnes et al., 1978) is used to assess the technical efficiency of DMUs. DEA measures efficiency of a DMU relative to a non-parametric estimate of the best-practice or efficient frontier constructed from the most efficient DMUs.

The main advantage of DEA is that it is able to handle multiple-input and multiple-output production situations and requires only an assumption of convexity of the production possibility set (Banker et al., 1984). However, despite its wide application, DEA has been criticized for its deterministic nature in that it does not impose an error term in the efficiency model and is therefore vulnerable to measurement error. In addition, DEA results are sensitive to the number of inputs and outputs chosen, particularly in small sample sizes, as a higher number of variables relative to the number of organizations measured could result in overestimates of efficiency scores (Charnes et al., 1994).

The DEA approach has several valuable properties, which make it amenable to this particular analysis. First, DEA is chosen because of its relative strength in dealing with small sample sizes compared to the regression based techniques (Jacobs et al., 2006). Second, DEA is better suited for contexts where data on input prices are not easily available as in many LMIC settings.

DEA models are broadly divided into output and input oriented models. Output oriented models determine the degree to which a firm can expand its output without changing its inputs. In contrast, input oriented models represent the degree to which a firm can reduce its input use without altering its outputs. In this study, technical efficiency of the health facilities was examined using an output oriented variable returns to scale (VRS) DEA. Output orientation was chosen based on the assumption that health facilities have more control over their outputs than their inputs. This is considered appropriate for public and NGO facilities in this setting, where resources are allocated centrally and therefore health facilities have no control over their inputs. In addition, it is expected that through integration, health providers will encourage the utilization of HIV services hence increasing output. Variable returns to scale assumes a production process in which the optimal mix of inputs and outputs is dependent on the scale of production.

The Farrell output-oriented measure of technical efficiency is obtained as:

Max $\theta_{0}$ Subject to:

$$\sum_{j=1}^{n}X_{ij}\lambda_{j}\;\leq \;X_{i0}\left(i=1\ldots \text{m}\right)$$

$$\sum_{j=1}^{n}Y_{rj}\lambda_{j}\;\geq \emptyset Y_{i0}\left(r=1\ldots \text{s}\right)$$

$$\sum_{j=1}^{n}\lambda_{j}=1$$

(1) $$\lambda_{j}\geq 0\left(j=1\ldots \text{n}\right)$$

where: $\theta_{0}$, the DEA efficiency score for DMU 0, is the maximum rate of proportional expansion in all outputs of DMU 0 given fixed levels of inputs; n is the number of DMUs; m is the number of inputs and s is the number of outputs; Y_r0_ is the amount of output r generated by unit 0 and X_i0_ is the amount of input i used by unit 0; ${\text{λ}}_{j}$ is the set of unique weights which DEA assigns to DMUj to maximize its output–input ratio. The technical efficiency of DMU 0 is obtained by calculating 1/ θ, and will be equal to 1 if the DMU is efficient and less than 1 if the DMU is inefficient when compared with other DMUs with similar output–input ratios.

Four different DEA models were estimated to measure efficiency of each health facility under one best practice frontier. Model 1 is a standard DEA model that only included the standard inputs (Staff FTE and floor space) and standard outputs (HIV and SRH outpatient visits). Model 2 included the same inputs and outputs, but also and incorporated quality as an output; Model 3 also included the same inputs and outputs but incorporated quality as an input variable. Model 4 included the same inputs and outputs but included the structural quality measure as an input and the process quality measure as an output. The results of the different models were used to assess how model specification affects the estimated technical efficiency of health facilities. Data was pooled for the two time periods and two countries and each observation treated as an independent realization of the data generating process. Pooling of data increases the sample size and provides more confidence in the precision of DEA estimates from the first stage analysis.

Given that the efficiency scores produced by DEA may be influenced by the presence of outliers especially with small samples, we investigated the presence of outliers following the method proposed by Wilson (Wilson, 1993). We graphically analyzed the log plot ratios to determine outliers and to investigate the effect of outliers on the efficiency scores; we removed the outlier facilities and estimated the efficiency scores without these units.

### 2.1 Determinants of efficiency

In the second stage, DEA efficiency scores (θ) are regressed against a set of environmental variables to investigate how these variables (integration in particular) affect the technical efficiency of health facilities. The truncated model is written as:

(2) $$0<\hat{\theta }=z_{i\;}\beta +\varepsilon_{i}\leq 1$$

where $\hat{{\text{θ}}_{\text{i}}}$ = ${\text{θ}}_{\text{i}}$ - bias (${\text{θ}}_{\text{i}}$) is the bias corrected estimator of technical efficiency and bias (${\text{θ}}_{\text{i}}$) is the bootstrap bias estimator of${\text{ θ}}_{\text{i}}$, ${\text{z}}_{\text{i }}$ is a vector of environmental variables which are hypothesized to have an effect on health facility efficiency, and β is the vector of parameters to be estimated.

Two methodological issues arise. First, DEA scores are sensitive to sampling variation and are upward biased by construction. Additionally, DEA efficiency estimates are serially correlated. The correlation arises in finite samples because the efficiency score of a DMU is estimated relative to the efficiencies of peer DMUs lying on the frontier.

To obtain unbiased beta coefficients and valid confidence intervals, a bootstrap simulation of the DEA scores obtained from the first stage was performed using FEAR (Frontier Efficiency Analysis with R) version 2.0 package in R. The bootstrap introduced by Efron (Efron, 1979) is a resampling method for statistical inference and is commonly used to estimate confidence intervals and to estimate bias and variance of an estimator. The bootstrap procedure produces bias-corrected efficiency scores between, but excluding 0 and 1 and results in a lower number of facilities with high efficiency scores (Mukherjee et al., 2010).

Since the regression residuals have a truncated distribution (because the DEA efficiency scores are bounded between 0 and 1), a truncated regression with a parametric bootstrap was performed. This produces robust regression coefficients and standard errors of the independent variables. The bias adjusted coefficients and the 95% bootstrap confidence interval are used to check the statistical significance of the estimated coefficients. The truncated regression model was performed in STATA version 12 and the steps of the double bootstrap procedure used follows Algorithm #2 of Simar and Wilson (Simar and Wilson, 2007).

### 2.2 Efficiency and quality

In addition to exploring the different ways to incorporate quality into the standard DEA model (as an additional input or output), we also examine quality and efficiency as two separate performance dimensions. Following Sherman and Zhu 2006 (Sherman and Zhu, 2006), we mapped facilities based on their quality and efficiency scores and divided them into four quadrants reflecting different definitions of high/low quality and high/low efficiency. Given that both the efficiency and quality scores are relative scores, we define high quality and high efficiency using the 75th percentile quality score of 5.3 and efficiency score of 0.645.

### 2.3 Sensitivity analysis

Due to the non-parametric nature of DEA, it is not possible to test model specifications or goodness of fit, as with parametric analysis. Given that DEA efficiency scores are sensitive to the inputs and output specification we assess the robustness of the estimated results by assessing the degree of correlation between the efficiency scores obtained from the four DEA models. In addition, we conducted the second stage regressions using the efficiency estimates obtained from each of the four different models estimated.

## 3 Data

Data on inputs and outputs used in this analysis were collected as part of a large non-randomized trial (Integra Initiative – ClinicalTrials.gov identifier: NCT01694862) from 40 health facilities in Kenya and Swaziland for 2008/2009 and 2010/2011. The Integra Initiative was aimed at strengthening the evidence base on the effect of integrating HIV and SRH services on a number of health outcomes and service costs (Warren et al., 2012). The sample consisted of two provincial hospitals, five district hospitals, six sub district hospitals, seventeen health centers, two public health units and eight sexual reproductive health non-governmental organization (NGO) clinics. The study sites were purposefully chosen from six priority regions with established programs based on previous operational research relationships with the Ministries of Health in Swaziland and Kenya. Randomized pair-wise matching was used to select intervention and comparison sites with similar characteristics based on a number of criteria. These criteria included high client load (more than 100 family planning (FP) clients per month), a minimum of two FP providers qualified in and currently providing FP services, a range of services (family planning, HIV counseling and testing and STI treatment) (Warren et al., 2012). The units of analysis are the HIV and SRH/maternal and child health (MCH) departments/public health unit (PHU) within health facilities.

Ethical approval for the Integra study was obtained from the Ethics Committee at the London School of Hygiene and Tropical Medicine (LSHTM) (approval no. 5436), from the Population Council Review Board (protocol nos. 443 and 444), from the Kenya Medical Research Institute (approval no. KEMRI/RES/7/3/1 protocol nos SCC/113 and SCC/114) and the Swaziland Scientific and Ethics Committee (approval nos. MH/599B and MH/599C).

### 3.1 Input and output variables

The choice of inputs and outputs for this analysis was guided by previous published efficiency literature, in which throughput measures are frequently used as outputs and human, capital and consumable resources are used as input variables (Jacobs et al., 2006). The production process of SRH and HIV services is characterized by labor and capital as inputs used to produce HIV and SRH outputs. Labor inputs were disaggregated into full time equivalents (FTE) for clinical staff and FTE for technical staff. Clinical staff included doctors, clinical officers and all cadres of nurses – senior nursing officer, nursing officer, registered nurses, enrolled nurses and nursing assistants. Technical staff included laboratory technologists and technicians, and pharmaceutical technologists and technicians, lay HIV counselors, peer educators and expert clients. Given that the inclusion of only salaried staff would underestimate the labor components of the health facilities, both labor categories included volunteer staff. Building space available for HIV and SRH services was used as a measure of capital input since HIV and SRH services require minimal equipment and because a reliable measure of the value of the equipment stock was rarely available.

The outputs used in this analysis represent the general services provided within the MCH/SRH and HIV units. These included: number of patients recorded receiving family planning (FP), cervical cancer (Ca Cx) screening, postnatal care (PNC), other MCH, HIV counseling and testing (HCT), treatment of sexually transmitted infections (STI), and HIV treatment and care services.

### 3.2 Environmental variables

Empirical work on efficiency measurement highlights the significance of organizational characteristics and differences in the production environments that could influence the efficiency of a DMU (Jacobs et al., 2006). The explanatory variables used in this analysis that reflect the structural differences in provision of health services, economic incentives and geographic and demographic factors include: the extent of integration, labor input mix, catchment population, facility ownership, geographic location, facility type and demographic factors such as demand for integrated SRH and HIV services.

Previous literature on the challenges of integration have noted that the extent of integration is dependent upon many factors that are beyond the control of the health facility such as staffing levels/labor input mix as well as population dynamics (Church et al., 2010, Kennedy et al., 2010). As such, in this analysis, integration is considered as a non-discretionary input rather than an input or output measure within the standard production model. Given that the rationale for integrating HIV and SRH services has been to improve the efficiency of delivering these services, we would therefore expect a positive relationship between the extent of integration and the technical efficiency.

Differences in labor input mix are measured by the percentage of clinical staff FTEs to the FTEs of other personnel (PROPCLS). We expect that the larger the proportion of clinical staff in a health facility, the more efficient on average that health facility would be. We also expect that the scale of operations is positively associated with technical efficiency and use the catchment population to control for scale of operations.

Agency and property rights theories both posit that private for profit and NGO facilities would be more efficient than government facilities due to differences in objectives, economic incentives, and control mechanisms (Tiemann et al., 2012). However, the empirical literature on the effect of ownership on hospital efficiency has reported mixed findings (Burgess and Wilson, 1996, Herr, 2008, Rosko, 1999, Rosko et al., 1995, Tiemann et al., 2012). A dummy variable (OWN = 1 if facility is a NGO clinic) is used to test whether public health facilities were less efficient compared to the NGO facilities. We expect a positive effect for NGO clinics.

A binary variable (HOSP = 1 if the facility is a hospital) is used to control for the facility type and test whether hospitals have efficiency advantages compared to smaller health facilities in the provision of integrated HIV and SRH services. Health facilities are classified as either hospitals (including provincial, district and sub district hospitals) or other health facilities (including health centers, public health units and SRH clinics). Assuming that facilities that operate at a large scale can realize greater technical efficiency due to increasing returns to scale, we would expect that hospitals would be more technically efficient than the smaller health centers and SRH clinics.

The location of a facility can be an important determinant of its efficiency. Urban location is hypothesized to have a positive effect on technical efficiency due to higher client volumes. A dummy variable (LOC = 1 if a facility is located in an urban area) is used to test whether the urban facilities were more efficient than their rural counterparts.

To control for differences in demand for different integrated services, we include the proportion of HIV related visits (PROPHIV) (total HIV visits/total HIV & SRH visits x 100). We expect that there will be an incentive for facilities with higher numbers of HIV visits to integrate services and therefore more technically efficient than facilities with fewer HIV visits. Finally, as data was collected at two time points (2008/9 and 2010/11), a dummy variable for 2010/2011 (YEARDUMMY) is used to control for the effects of time. A summary of definitions and descriptive statistics of input, outputs and environmental variables used in the analysis is provided in Table 1.

### 3.3 Quality of health service variables

The measurement of quality of health service is based on the standard framework provided by Donabedian (Donabedian, 1988) and incorporates structural, interpersonal and technical attributes of quality. The structural attributes of quality assessed included availability of infrastructure and equipment, commodities and management practices (availability of guidelines/standards and information and education (IEC) materials). These were assessed through a health facility inventory assessment administered at each study facility and were used to ascertain the availability of the appropriate inputs. The interpersonal and technical aspects of quality, were assessed through observations of the client–provider interactions at each health facility (Warren et al., 2012). Interpersonal aspects of quality refer to the interaction between the patient and the health provider while the technical aspects refer to how well medical knowledge is applied to diagnosis and treatment of the medical problem. The quality attributes used in this analysis are presented in Table 2.

A score was provided for each attribute and a composite quality score was generated for each health facility by combining the structural and process indicators of quality into a single score using principal component analysis (PCA). PCA is a statistical technique which decomposes data with correlated values into a set of uncorrelated (orthogonal) variables (Jobson, 1992). The uncorrelated variables are referred to as principal components or factors and are a linear combination of the standardized values of the original variables used in the definition of the index. The weight given to each of the components corresponds to its statistical correlation with the latent dimension that the index is measuring. Using the factor scores from the first principal component as weights, an index of quality of health service is constructed for each health facility with a mean equal to zero and a standard deviation equal to one. A summary of the quality of care scores and results from the PCA by country is presented in the Supplementary Appendix (Insert link to online file A).

### 3.4 Measures of integration

Although HIV/SRH integration has been a national policy in both Kenya and Swaziland since 2009, the extent of integration varies widely across facilities with varying degrees of structural and functional integration. The extent of facility integration is measured in this paper using two indices of integration: structural (STINT) and functional integration (FUNINT) indices developed using latent variable techniques incorporating expert opinions. The variables used to develop the structural index of integration were: number of HIV/STI services available within the entire facility; number of HIV/STI services available within the MCH/PHU; number of services provided per clinical staff; and the number of services provided in each consultation room.

The functional index of integration focused on an assessment of service utilization patterns in each of the study facilities. The variables used to develop this index were: the average number of services accessed across days of the week; the average number of services accessed in single consultations; the average number of services accessed in single visits. Data used to develop these variables were obtained from facility register data, other records review and observations of staff, as part of the larger Integra Initiative (Warren et al., 2012).

## 4 Results

### 4.1 Technical efficiency

Table 3 presents the uncorrected and bias corrected mean technical efficiency scores obtained from the four DEA models. Overall, the DEA results indicate considerable variation in efficiency scores between the different model specifications. Bias corrected mean efficiency scores range from 0.45 (standard DEA model 1 with no quality measure), 0.65 (model 2 with quality as an output); 0.49 (model 3 with quality as input) and 0.28 (model 4 with structural quality measure as input and process quality measure as output). These results suggest that there is a high degree of inefficiency in the health facilities providing integrated HIV and SRH services.

Table 3 also compares the standard DEA model (model 1) with the models 2, 3 and 4 where quality is included in the DEA model. In this case, we found that the mean bias corrected technical efficiency scores increase when quality is included in the DEA model as an output, rising from 0.45 to 0.65. Comparing models 1 (standard DEA model with no quality) and 3 (model with quality as input), the mean bias corrected technical efficiency scores increase, although marginally, from 0.45 to 0.49 when quality is considered as an input. These results are consistent with other studies incorporating quality as either an input or output which find that technical efficiency increases after the inclusion of quality measures in the standard DEA. When models 1 and 4 are compared, the mean bias corrected technical efficiency scores decrease when the structural and process measures of quality are included as inputs and output variables respectively.

The degree of correlation between the efficiency scores for each DMU obtained using the standard DEA model (model 1) and the scores obtained with quality included (models 2, 3 and 4) was examined using the Spearman rank correlation coefficient. The degree of correlation between the efficiency scores for each health facility obtained from the models with only the technical variables and those with both technical and quality variables were 0.713 (models 1 and 2), 0.818 (models 1 and 3) and 0.75 (models 1 and 4) which shows that the results are to some degree sensitive to the DEA model specification.

The analysis of outliers identified four outliers. We found a high correlation coefficient.

(0.925 p < 0.001) between the initial efficiency scores and those when the four outliers are removed. This suggests that the removal of the outlier facilities did not have a substantial effect on our efficiency scores.

**Fig. 1:**
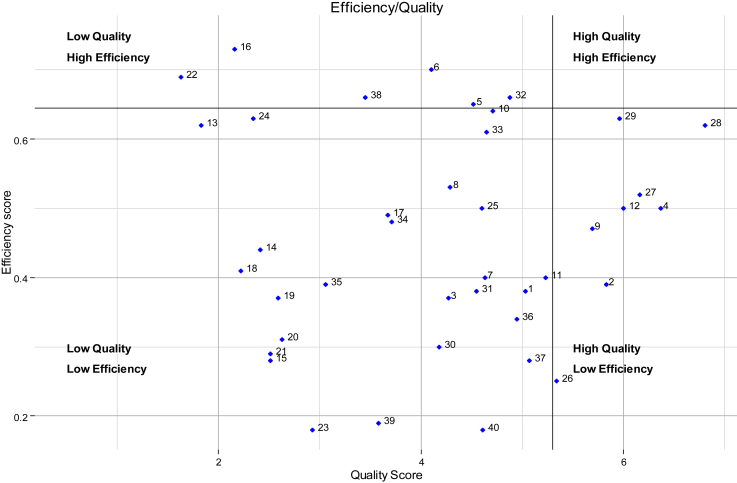
Technical efficiency and quality distribution of health facilities.

Fig. 1 descriptively maps the composite quality of care scores against the technical efficiency scores with no quality (model 1) for each health facility. The graph is separated into four segments to reflect the ways in which quality and efficiency may be defined - high quality and high efficiency facilities (HQ-HE); high quality and low efficiency facilities (HQ-LE); low quality and low efficiency facilities (LQ-LE); and low quality and high efficiency facilities (LQ-HE). The best practice is defined as high quality, high efficiency (HQ-HE). Although fourteen of the forty facilities were mapped in the low quality/high efficiency (LQ/HE) and high quality/low efficiency (HQ/LE) quadrants, the majority (26) of the facilities were mapped as low quality/low efficiency. Although these results are limited in that only one third of sites appear to demonstrate a trade-off, this may be sufficient to support the DEA results from models 2 and 3 above, where including a composite quality score measure into the standard DEA reduces variability across sites. More generally the low efficiency and quality scores across the study sample are cause for concern.

### 4.2 Determinants of technical efficiency

The dependent variables in the second stage-truncated regressions are the bias corrected technical efficiency scores obtained using DEA models 1–4. A positive (negative) coefficient indicates a positive (negative) marginal effect on technical efficiency. Table 4 summarizes the results of the bootstrapped truncated regressions and shows that the mean variance inflation factor is 2.81. This implies that the models do not suffer from multi-collinearity problems. Correlations between explanatory variables were also not statistically significant. Overall, the results of Model 2 show a slight improvement in the statistical significance of the estimated coefficients relative to the other models.

When the structural aspects of integration were disaggregated, the regression results show that the number of HIV/STI services in the MCH unit and the number of HIV/STI services provided per room are the only statistically significant measures of integration across all four models. No significant effect was found for the functional index of integration or the number of additional HIV/STI services within the facility and number of additional HIV services provided per clinical staff.

Contrary to expectation, we found that public health facilities and other health facilities (health centers and clinics) had significantly higher levels of technical efficiency compared to the NGO facilities and hospitals. Surprisingly we also found a negative and significant effect for the proportion of clinical staff on technical efficiency of health facilities.

## 5 Discussion and conclusions

The results of the DEA indicate low estimates of technical efficiency across all model specifications suggesting a substantial level of inefficiency exists across integrated HIV/SRH services in the facilities studied. Our results also show a weak effect of the extent of integration on efficiency of HIV and SRH services and suggest a complex relationship between integration and efficiency.

Although we found a positive significant effect of the number of HIV/STI services in the MCH unit on technical efficiency, no statistically significant effect was found for the functional integration measure. This result is puzzling at first glance, however a closer examination suggests a complex relationship between availability of services and actual delivery of services. Firstly, structural and functional integration may not occur in tandem. Recent findings by Mayhew et al. (Mayhew et al., 2016) show that while health facilities may have the capacity to integrate services, this may not necessarily result in receipt of integrated services by clients. Indeed, it is possible that facilities may not be able to deliver integrated services due to other factors related more generally to the health system. Such inhibiting factors identified across a wide range of settings may include poor facility management and supervision; staff shortages, high turnover, and inadequate staff training; inadequate infrastructure, equipment, and commodity supply; as well as client barriers to service utilization, including low literacy and acceptance of services (Kennedy et al., 2010). Secondly, while having increased services on offer may increase the number of services provided per input, providing services in one visit for one client may require complex management and may reduce efficiency.

The finding of a negative and significant effect of the number of HIV/STI services per clinical room suggests that integration of HIV/STI services in one room may reduce technical efficiency of service delivery. Although puzzling, observations at the health facilities support this finding, since where providers have multiple rooms available for service delivery, providers are better able to manage their client flow as they can provide multiple services simultaneously. For example, they can provide HIV counselling and testing in one room and then move on to another room to provide another service while the other client is waiting for their HIV results.

The significance of ownership and facility type across all models strengthens the evidence (Jacobs et al., 2006) that there are certain environmental characteristics of health facilities that affect the technical efficiency of HIV and SRH service delivery. The robustness of these findings can be attributed to their consistency across all four models. However, the interpretation that public health facilities and health centers/clinics operate at higher technical efficiency relative to their NGO and hospital counterparts should be made with caution. Both the NGO clinics and the large hospitals handle relatively more complicated cases thus providing more sophisticated outputs (e.g. long term family planning methods, and pap smears for cervical screening) an element which is not captured by the technical outputs when described as uniform services. A recommendation to promote decentralization of services to smaller health facilities based on technical efficiency results obtained without taking into account this difference in case mix would therefore be misleading.

The negative significant result of the proportion of clinical staff from total staff on technical efficiency while surprising may be plausible because clinical staff are better trained and therefore spend more time with a client which lowers their technical efficiency. While this may be considered as a proxy for quality of care, it may also suggest lack of good management in allocating resources effectively across services. The negative and significant coefficient of the proportion of HIV services provided in the facility may be attributed to the fact that HIV services generally take longer to provide and are therefore associated with higher resource input lowering the technical efficiency of health facilities.

One of the main strengths of this study is that it incorporates quality measures into the exploration of the association between integration and technical efficiency. As stated earlier, few studies have considered quality issues when estimating efficiency, even though quality considerations are relevant to ensure that efficiency gains are not made at the expense of quality of health services. From the analysis of quality and efficiency as separate dimensions of performance, we find that majority of the study facilities exhibit low efficiency and low quality of care, rather than a trade-off between them. Our results show that facilities that perform poorly transforming inputs into outputs also perform poorly in terms of transforming structural aspects of quality into quality outputs. Factors that drive poor non-quality adjusted efficiency may therefore also drive poor quality. In particular, the underlying data suggests that facilities scoring poorly on both fronts also demonstrate a lack of availability of resources (including basic infrastructure, equipment and commodity supply). This challenges the viewpoint that inefficiency may be primarily due to over resourcing (particularly of HIV services), but rather is a function of poor support to some facilities to implement expanded service delivery.

A number of limitations of this analysis due to data quality and measurement error, typical of the challenges of efficiency and quality measurement in LMIC settings, should be noted. First, both input and output data were in part derived from routine data sources; and even though these were checked using both observation and client survey data, there remains room for error. Second, given the lack of client classification systems according to the complexity of the HIV and/or SRH service and resource consumption in both contexts, case mix effects were also not fully considered in this analysis. Further research on the extent to which detailed SRH case mix influences efficiency and how to incorporate these effects into a model is needed. Third, in relation to quality measurement, the study considered only structural and process aspects of quality. Future research on integrated HIV and SRH services would benefit from the incorporation of outcome measures that denote the effects of care on the health status of patients, which was difficult to obtain in this context. These limitations may limit the generalizability of the results of the study across settings.

Finally, the validity of these results may be challenged as the regression model does not account for endogeneity. In this particular context, it is likely that there may be an issue of reverse causality between integration and technical efficiency and the direction of causality is not clear. In practice, it is possible that a higher degree of integration within a facility can improve efficiency but also that health facilities that are efficiently managed are better able to integrate services more readily. One of the ways to address the issue of endogeneity in the econometric literature has been the use of instrumental variable approaches. A limitation of this study is that the small panel dataset available could not provide for a valid instrument necessary to correct for the potential endogeneity of integration.

Our detailed findings cannot be generalized to other settings as an indication of current levels of inefficiency in SRH/HIV. However, broadly our findings challenge the assumption and policy claims that either integration can improve efficiency, or that there is a strong trade-off between quality and efficiency. Instead we find complex results that suggest that many facilities are performing poorly on both efficiency and quality fronts. This finding suggests that even when structural interventions aimed at improving integration are implemented, these may fail to result in improved outputs. Policy makers may therefore need to examine not just the technical aspects of facility improvement, but also change management, management capacity and the under-resourcing of facilities more generally to address the extent of poor performance, when implementing policies such as integration (Hope et al., 2014). While technically complex, approaches such as this study that consider measures of quality, can help policy makers target which facilities to support. Finally, there are number of research gaps remaining. Pragmatic trials of organizational change interventions around integration are required to learn more about which approaches work and do not; and further DEA studies across other settings in sub Saharan Africa are required to further confirm and explore the complex relationship between quality and efficiency.

This paper applied recent methodological advancements in health care efficiency analysis, and provided some important first insights not only into the technical efficiency of health facilities providing integrated HIV and SRH services but also into some of the determinants of technical efficiency. The empirical results reveal a high degree of inefficiency in the health facilities providing integrated HIV and SRH services. The number of additional HIV services in the MCH, public ownership and facility type, have a positive significant effect on technical efficiency. However, the number of HIV and SRH services provided in the same clinical room, proportion of clinical staff to overall staff, proportion of HIV services provided, and rural location had negative significant effects on technical efficiency.

This paper also aims to provide insight on how quality could be incorporated into efficiency measurement studies in the LMIC context. The results of the analysis show that relying on efficiency measures without controlling for quality of care may provide misleading results and should not be used to infer potential efficiency gains, pointing to a need for further work in this under-researched area. Importantly, the results show the performance of health facilities in relation to quality and efficiency within the context of integrated HIV and SRH services highlighting the need to improve both quality and efficiency of service delivery.

## Figures and Tables

**Table 1:** Definition and summary statistics of variables used in the study for 2008–09 and 2010–11.

Variable	Measurement	2008–09 (n = 40) mean [SD]	2010–11 (n = 40) mean [SD]	2008-1011 (n = 80) mean [SD]
**Inputs**				
Clinical FTE	Number of Doctor, clinical officer and nurse FTEs	8 (5.38)	10 (7.04)	9 (6.35)
Non clinical FTE	Number of Lab technologist, pharmaceutical technologist, lay counsellor, and other admin staff FTEs	8 (5.63)	11 (6.12)	9 (6.01)
Unit size	Square footage available for HIV and SRH services	194.20 (147.61)	214.00 (158.26)	204 (152.39)
**Outputs**				
Ca Cx visits	Total annual visits for cervical cancer screening	163 (277.34)	244 (430.89)	203 (362.31)
FP visits	Total annual visits for family planning	3505 (2949.04)	4270 (4140.37)	3887 (3592.31)
PNC visits	Total annual visits for post-natal care	527 (812.87)	848 (900.27)	687 (867.39)
HCT visits	Total annual visits for HIV counseling and testing	1867 (1596.08)	3474 (3549.17)	2670 (2851.38)
STI visits	Total annual visits for treatment of sexually transmitted infections	242 (599.59)	313 (735.95)	277 (667.93)
HIV visits	Total annual visits for HIV treatment	2868 (7145.13)	4627 (12108.25)	6696 (10527.47)
Other visits	Total annual other MCH visits	11808 (9886.75)	12600 (13095.74)	12204 (11535.94)
SRH visits	Total annual aggregated Sexual and Reproductive Health visits (FP, PNC, Ca Cx and STI)	4196 (3593.02)	5363 (4635.37)	4779 (4162.38)
HCT/HIV visits	Total annual aggregated HIV visits (HCT & HIV treatment)	4977 (7723.53)	8414 (12601	6696 (10527.47)
Structural quality score	Structural quality index score	2.04 (1.49)	4.35 (2.03)	3.2 (2.11)
Process quality score	Process quality index score	3.13 (1.76)	3.56 (1.37)	3.35 (1.58)
QOC score	Composite index score for structural and process quality indicators	2.92 (1.71)	5.31 (2.09)	4.14 (2.25)
**Environmental variables**				
HIVSTI FAC	HIV/STI services provided in the facility	6.5 (1.25)	6.7 (0.91)	6.64 (1.09)
HIVSTI MCH	HIV/STI services provided in the MCH unit	2.44 (1.18)	2.45 (1.10)	2.45 (1.14)
HIVSTICS	HIV/STI service provided per clinical staff	1.86 (0.98)	1.76 (0.96)	1.81 (0.97)
HIVSTIR	HIV/STI services provided per room	1.37 (0.92)	1.35 (0.92)	1.36 (0.92)
FUINT	Functional integration score	1.24 (0.93)	1.29 (0.97)	1.27 (0.94)
LN(POP)	Logarithm of catchment population	11.40 (1.51)	11.31 (1.53)	11.35 (1.51)
PROPHIV	Proportion of HIV related visits of total HIV/SRH visits	0.20(0.18)	0.29 (0.19)	0.24(0.19)
PROPCS	Proportion of clinical staff to other staff	0.49(0.15)	0.48 (0.15)	0.48(0.15)
PUBLIC	Government health facilities (binary variable 1,0)	0.80 (−)	0.80 (−)	0.80 (−)
Other Facilities	Health centers and clinics (binary variable 1,0)	0.67 (−)	0.67 (−)	0.67 (−)
RURAL	Rural facility (binary variable 1,0)	0.58 (−)	0.58 (−)	0.58 (−)

**Table 2:** Summary of quality attributes.

Indicator	Definition of indicators
**Structural**	
Physical infrastructure	Availability of amenities: shaded waiting area, private space for FP examination, private space for ANC/PNC examination, source of clean water, electricity, clean toilets, reliable lighting, infection prevention buckets, heater, chlorine for processing equipment
Equipment availability	Availability of the following equipment: Spotlight or flashlight, exam couch, waste receptacle, sharps container, electric hand dryer or single use towels, functional blood pressure machine, stethoscope, functional weighing scale for babies, functional weighing scale for adults, speculum, tenaculum, uterine sound, working autoclave/sterilizer, cleaning solution, Trocar, Kidney dishes, sponge holding forceps, foetal scope
FP commodities	FP methods available: combined pill, progestin only, emergency contraceptive, injectables, female condoms, male condoms, IUCD, Cycle beads, hormonal implants, female sterilization, male sterilization
Reagents	Testing reagents available: HIV-1 reagents, HIV-2 reagents, UNIGOLD, Determine, TB test, pregnancy tests, UTI
General supplies	General supplies available: needles and syringes, insecticide treated nets, specimen bottles for urine, specimen pots for sputum, blood specimen pots, slides for MPS, vinegar, Acetic acid, iodine, lugols, IV giving sets, blood giving sets, normal saline IV, Sodium lactate IV solution, Dextrose IV solution, Ringers lactate IV solution, Water for injection
Staff training	Whether any of the staff has received training in the following: PMTC, HIV counseling and testing, HIV rapid tests and controls, STI syndromic management, syphilis screening for RPR test, balanced counseling strategy plus, counseling for prevention of STIs, counseling for prevention of HIV/AIDS, medical management of HIV infected clients, Screening for TB in pregnancy, FANC, management of labor, basic care of newborns, infant feeding counseling, family planning, contraceptive technology updates, IMCI, post-natal care for baby, screening for cancer using VIA/VILLI.
IEC materials	Number of visual aids for teaching available in the counseling rooms: FP methods, STIs, HIV/AIDS, PMTCT for HIV, balanced counseling strategy cards; condom model; FP ANC and PNC posters, danger signs in postpartum period for mother and babies.
Guidelines, policies and standards	Availability of protocols, guidelines and standards on: FP policy, FANC orientation, essential obstetric care, standard maternity care, PNC guidelines, STI syndromic management guidelines, PMTCT guidelines, ART guidelines, clinical manual for ARV providers, HIV testing guidelines, pre/post counseling protocol for HIV, TB treatment protocol.
**Process**	
Privacy and confidentiality assured	Does the provider see client in privacy and assure the client of confidentiality
Clients questions answered	Does the provider ask if client has understood information and encourage client to ask questions?
Reproductive history	Provider discussed the following: age, marital status, pregnancy status, number of pregnancies, fertility desires, breastfeeding status, desired timing of next birth, date of last menses, previous use of FP, HIV serostatus, history of medical conditions
Family planning procedure	Does the provider discuss the following: explain how method works, advantages and disadvantages, how to use method, ensuring effectiveness, possible side effects, management of side effects, possibility of changing method, emergency contraception
HIV/STI risk assessment	Does the provider discuss STIs and HIV risk factors with clients: multiple partners, STIs, unprotected sex, knowledge of partners' status and HIV counseling and testing?

**Table 3:** Uncorrected and bias corrected efficiency scores results from the first stage DEA with bootstrap.

		Model 1: No quality			Model 2: Quality as an output variable			Model 3: Quality as an input variable			Model 4: Structural quality as input and process quality as output		
Uncorrected DEA scores	Bias corrected eff scores		Uncorrected DEA scores	Bias corrected eff scores		Uncorrected DEA scores	Bias corrected eff scores		Uncorrected DEA scores	Bias corrected eff scores	
Year	N	Mean	Mean	SD	Mean	Mean	SD	Mean	Mean	SD	Mean	Mean	SD
Pooled	80	0.75	0.45	0.19	0.84	0.65	0.15	0.79	0.49	0.17	0.60	0.22	0.26
2009	40	0.68	0.42	0.19	0.79	0.62	0.16	0.74	0.46	0.19	0.67	0.34	0.20
2011	40	0.82	0.49	0.19	0.89	0.68	0.14	0.86	0.52	0.16	0.63	0.28	0.24

**Table 4:** Determinants of technical efficiency.

Variables	Model 1 (No quality) β	Model 2 (quality as output) β	Model 3 (quality as input) β	Model 4 (structural quality -input/process quality – output) β
HIVSTIFAC	−0.014	−0.008	−0.0214	0.028
HIVSTI MCH	0.083*	0.050*	0.085**	0.095**
HIVSTICS	0.039	0.009	0.029	0.017
HIVSTIR	−0.096**	−0.085**	−0.078**	−0.046**
FUINT	−0.004	0.007	0.004	0.020
LN(POP)	0.004	−0.009	0.006	0.003
PROPHIV	−0.103	−0145*	−0.119	−0.035
PROPCS	−0.413**	−0.058	−0.345**	−0.070
PUBLIC	0.353**	0.161**	0.322***	0.069
Other facilities	0.190**	0.124**	0.158**	0.119**
RURAL	−0.062	−0.164***	−0.045	−0.038
Year2011	0.077*	0.069**	0.073**	0.0456**
Sigma	0.178***	0.135***	0.148***	0.157***
Log-likelihood	25.57	46.42	35.88	34.71
Mean VIF	2.81			

Dependent variable: DEA bias-corrected efficiency scores from models 1–4.

^∗∗∗^, ^∗∗^,^∗^ denote significance at 1%, 5% and 10% levels. Confidence intervals obtained from 1000 bootstrap interactions.

## References

[bib1] Arocena P., García-Prado A. (2007). Accounting for quality in the measurement of hospital performance: evidence from Costa Rica. Health Econ..

[bib2] Au N., Hollingsworth B., Spinks J. (2014). Measuring the efficiency of health services in lower-income countries: the case of papua New Guinea. Dev. Policy Rev..

[bib3] Banker R.D., Charnes A., Cooper W.W. (1984). Some models for estimating technical and scale inefficiencies in data envelopment analysis. Manag. Sci..

[bib4] Burgess J.F., Wilson P.W. (1996). Hospital ownership and technical inefficiency. Manag. Sci..

[bib5] Charnes A., Cooper W., Lewin A., Seiford L. (1994). Data Envelopment Analysis: Theory, Methodology and Applications.

[bib6] Charnes A., Cooper W., Rhodes E. (1978). Measuring the efficiency of decision making units. Eur. J. Oper. Res..

[bib7] Church K., de Koning K., Hilber A.M., Ormel H., Hawkes S. (2010). Integrating sexual health services into primary care: an overview of health systems issues and challenges in developing countries. Int. J. Sex. Health.

[bib8] Cordero Ferrera J., Cebada E., Murillo Zamorano L. (2013). The effect of quality and socio-demographic variables on efficiency measures in primary health care. Eur. J. Health Econ..

[bib9] Donabedian A. (1988). The quality of care: how can it be assessed?. JAMA.

[bib10] Dudley L., Garner P. (2011). Strategies for integrating primary health services in low-and middle-income countries at the point of delivery. Cochrane Database Syst. Rev..

[bib11] Efron B. (1979). Bootstrap methods: another look at the jackknife. Ann. Stat..

[bib12] García F., Marcuello C., Serrano D., Urbina O. (1999). Evaluation of efficiency in primary health care centres: an application of data envelopment analysis. Financ. Acc. abil. Manag..

[bib13] Herr A. (2008). Cost and technical efficiency of German hospitals: does ownership matter?. Health Econ..

[bib14] Hollingsworth B. (2008). Efficiency Measurement in Health and Healthcare/Bruce Hollingsworth and Stuart J. Peacock.

[bib15] Hope R., Kendall T., Langer A., Bärnighausen T. (2014). Health systems integration of sexual and reproductive health and hiv services in sub-Saharan Africa: a scoping study. J. Acquir. Immune Defic. Syndromes (1999).

[bib16] Ickovics J. (2008). “Bundling” HIV prevention: integrating services to promote synergistic gain. Multiple Health Behav. Change (MHBC) Res..

[bib17] Jacobs R., Smith P.C., Street A. (2006). Measuring Efficiency in Health Care: Analytic Techniques and Health Policy.

[bib18] Jobson J.D. (1992). Principal components, factors and correspondence analysis. Appl. Multivar. Data Anal..

[bib19] Kennedy C.E., Spaulding A.B., Brickley D.B., Almers L., Mirjahangir J., Packel L., Osborne K. (2010). Linking sexual and reproductive health and HIV interventions: a systematic review. J. Int. AIDS Soc..

[bib20] Kisubi W., Farmer F., Sturgis R. (1997). An African Response to the Challenge of Integrating STD/HIV-aids Services into Family Planning Programs.

[bib21] Mayhew S.H., Ploubidis G.B., Sloggett A., Church K., Obure C.D., Birdthistle I. (2016). Innovation in evaluating the impact of integrated service-delivery: the integra indexes of HIV and reproductive health integration. PLoS One.

[bib22] Mukherjee K., Santerre R., Zhang N.J. (2010). Explaining the efficiency of local health departments in the U.S.: an exploratory analysis. Health Care Manag. Sci..

[bib23] Murray C.J.L., Anderson B., Burstein R., Leach-Kemon K., Schneider M., Tardif A., Zhang R. (2011). Development assistance for health: trends and prospects. Lancet.

[bib24] Nedelea I., Fannin J. (2013). Technical efficiency of critical access hospitals: an application of the two-stage approach with double bootstrap. Health Care Manag. Sci..

[bib25] Nyman J.A., Bricker D.L., Link D. (1990). Technical efficiency in nursing homes. Med. Care.

[bib26] Rosenman R., Friesner D. (2004). Scope and scale inefficiencies in physician practices. Health Econ..

[bib27] Rosko M.D. (1999). Impact of internal and external environmental pressures on hospital inefficiency. Health Care Manag. Sci..

[bib28] Rosko M.D., Chilingerian J.A., Zinn J.S., Aaronson W.E. (1995). The effects of ownership, operating environment, and strategic choices on nursing efficiency. Med. Care.

[bib29] Salinas-Jiménez J., Smith P. (1996). Data envelopment analysis applied to quality in primary health care. Ann. Op. Res..

[bib30] Sherman H.D., Zhu J. (2006). Benchmarking with quality-adjusted DEA (Q-DEA) to seek lower-cost high-quality service: evidence from a U.S.bank application. Ann. Op. Res..

[bib31] Simar L., Wilson P.W. (2007). Estimation and inference in two-stage, semi-parametric models of production processes. J. Econ..

[bib32] Sobczak A. (2002). Opportunities for and constraints to integration of health services in Poland. Int. J. Integr. Care.

[bib33] Tiemann O., Schreyögg J., Busse R. (2012). Hospital ownership and efficiency: a review of studies with particular focus on Germany. Health Policy.

[bib34] UNAIDS (2013). Global Report: UNAIDS Report on the Global AIDS Epidemic 2013.

[bib35] Wagner J.M., Shimshak D.G., Novak M.A. (2003). Advances in physician profiling: the use of DEA. Socio-Economic Plan. Sci..

[bib36] Warren C., Mayhew S., Vassall A., Kimani J.K., Church K., Obure C.D., Watts C. (2012). Study protocol for the integra initiative to assess the benefits and costs of integrating sexual and reproductive health and HIV services in Kenya and Swaziland. BMC Public Health.

[bib37] Wilson P.W. (1993). Detecting outliers in deterministic nonparametric frontier models with multiple outputs. J. Bus. Econ. Stat..

