# Supplementary material for: Does integration of HIV and sexual and reproductive health services improve technical efficiency in Kenya and Swaziland? An application of a two-stage semi parametric approach incorporating quality measures
Source: Soc Sci Med. 2016 Feb;151:147–56. doi: 10.1016/j.socscimed.2016.01.013 (PMC4774477; doi:10.1016/j.socscimed.2016.01.013)
Supplement: Supplementary file 1 [file mmc1.docx]

Online File A: Summary of quality of care scores and results by country from principal components analysis

| **Variable description** | **Overall index** | **Kenya** | | | **Swaziland** | | |
| --- | --- | --- | --- | --- | --- | --- | --- |
|  |  |  |  |  |  |  |  |
|  | **Factor score** | **Mean** | **Std Dev.** | **Factor score** | **Mean** | **Std Dev.** | **Factor score** |
| **STRUCTURE** |  |  |  |  |  |  |  |
| **Infrastructure and equipment** |  |  |  |  |  |  |  |
| Physical Infrastructure | ***0.38*** | 5.3 | 5.61 | *0.39* | 6.7 | 1.03 | *0.45* |
| Equipment availability | ***0.31*** | 15.5 | 5.28 | *0.33* | 15.8 | 3.14 | *0.43* |
| **Commodities** |  |  |  |  |  |  |  |
| FP commodities | ***0.34*** | 8.3 | 2.77 | *0.37* | 8.2 | 1.19 | *-0.17* |
| Reagents | ***0.23*** | 6.9 | 5.07 | *0.25* | 2.2 | 1.00 | *-0.44* |
| General supplies | ***0.29*** | 8.8 | 7.09 | *0.30* | 5.6 | 2.76 | *0.45* |
| **Management** |  |  |  |  |  |  |  |
| Staff training | ***0.29*** | 9.3 | 7.36 | *0.33* | 11.3 | 3.84 | *0.18* |
| IEC materials | ***0.34*** | 7.0 | 8.61 | *0.39* | 12.1 | 6.43 | *0.32* |
| Guidelines and standards | ***0.38*** | 5.1 | 5.69 | *0.42* | 8.2 | 3.00 | *0.22* |
| **PROCESS** |  |  |  |  |  |  |  |
| **Interpersonal** |  |  |  |  |  |  |  |
| Privacy/confidentiality | ***0.23*** | 1.3 | 0.64 | *0.42* | 1.1 | 0.31 | *-0.05* |
| Clients questions answered | ***0.25*** | 1.3 | 0.64 | *0.47* | 1.3 | 0.47 | *0.46* |
| **Technical** |  |  |  |  |  |  |  |
| Reproductive history | ***-0.03*** | 5.0 | 2.18 | *0.41* | 6.3 | 1.59 | *0.48* |
| Family planning procedure | ***0.19*** | 3.1 | 2.23 | *0.49* | 2.4 | 1.79 | *0.53* |
| HIV/STI risk assessment | ***0.05*** | 1.5 | 1.28 | *0.44* | 1.4 | 1.54 | *0.52* |
